# Notifiable disease reporting among public sector physicians in Nigeria: a cross-sectional survey to evaluate possible barriers and identify best sources of information

**DOI:** 10.1186/s12913-014-0568-3

**Published:** 2014-11-13

**Authors:** Kathryn E Lafond, Ibrahim Dalhatu, Vivek Shinde, Ekanem E Ekanem, Saidu Ahmed, Patrick Peebles, Mwenda Kudumu, Milele Bynum, Kabiru Salami, Joseph Okeibunor, Pamela Schwingl, Anthony Mounts, Abdulsalami Nasidi, Diane Gross

**Affiliations:** Influenza Division, Centers for Disease Control and Prevention, Atlanta, GA USA; Centers for Disease Control and Prevention, Abuja, Nigeria; Federal Ministry of Health, Abuja, Nigeria; Social and Scientific Systems, Inc, Silver Spring, MD USA; World Health Organization, Geneva, Switzerland; Nigeria Centre for Disease Control, Abuja, Nigeria

**Keywords:** Nigeria, Health knowledge, Attitudes, Practice, Disease notification, Influenza A virus, H5N1 subtype

## Abstract

**Background:**

Since 2001, Nigeria has collected information on epidemic-prone and other diseases of public health importance through the Integrated Disease Surveillance and Response system (IDSR). Currently 23 diseases are designated as “notifiable” through IDSR, including human infection with avian influenza (AI). Following an outbreak of highly pathogenic avian influenza A(H5N1) in Nigerian poultry populations in 2006 and one laboratory confirmed human infection in 2007, a study was carried out to describe knowledge, perceptions, and practices related to infectious disease reporting through the IDSR system, physicians’ preferred sources of heath information, and knowledge of AI infection in humans among public sector physicians in Nigeria.

**Methods:**

During November to December 2008, 245 physicians in six Nigerian cities were surveyed through in-person interviews. Survey components included reporting practices for avian influenza and other notifiable diseases, perceived obstacles to disease reporting, methods for obtaining health-related information, and knowledge of avian influenza among participating physicians.

**Results:**

All 245 respondents reported that they had heard of AI and that humans could become infected with AI. Two-thirds (163/245) had reported a notifiable disease. The most common perceived obstacles to reporting were lack of infrastructure/logistics or reporting system (76/245, 31%), lack of knowledge among doctors about how to report or to whom to report (64/245, 26%), and that doctors should report certain infectious diseases (60/245, 24%). Almost all participating physicians (>99%) reported having a cell phone that they currently use, and 86% reported using the internet at least weekly.

**Conclusions:**

Although the majority of physicians surveyed were knowledgeable of and had reported notifiable diseases, they identified many perceived obstacles to reporting. In order to effectively identify human AI cases and other infectious diseases through IDSR, reporting system requirements need to be clearly communicated to participating physicians, and perceived obstacles, such as lack of infrastructure, need to be addressed. Future improvements to the reporting system should account for increased utilization of the internet, as well as cell phone and email-based communication.

**Electronic supplementary material:**

The online version of this article (doi:10.1186/s12913-014-0568-3) contains supplementary material, which is available to authorized users.

## Background

Nigeria, the most populous country in Africa with approximately 155 million people [1], has an estimated 140 million poultry, nearly 60% of which are raised in backyard flocks [2]. Highly pathogenic avian influenza A(H5N1) (HPAI) in domestic birds was first reported in Africa at a commercial farm in Kaduna state, Nigeria in February 2006 [2]. In response to this outbreak, millions of chickens were culled. Between 2006 and 2011, Nigeria reported 65 HPAI outbreaks in birds [3], with identification of virus from 3 distinct lineages [4], suggesting ongoing transmission across and within national borders. Subsequent to the introduction of HPAI in birds in 2006, a number of response activities took place including physician training workshops and awareness campaigns, and the development of state-level rapid response teams.

Another response to the identification of HPAI in birds was the addition of avian influenza (AI) human infections to the list of notifiable diseases in Nigeria, requiring it to be reported through the Integrated Disease Surveillance and Response system (IDSR). The current Nigerian IDSR system was established in 2001 [5], with 23 diseases designated as “notifiable” [6]. These diseases are categorized as: epidemic-prone (cholera, measles), targets for eradication/elimination (poliomyelitis, dracunculiasis), and other diseases of public health importance (malaria, AI). Through the IDSR system, notifiable diseases are identified at all public primary, secondary, and tertiary care facilities nationwide, and follow a reporting chain from the facility, to local government authorities (LGAs), to state-level Ministry of Health, and finally to the Federal Ministry of Health (FMOH), who then analyze and disseminate IDSR data.

The first confirmed human case of AI in the World Health Organization’s African Region (WHO-AFRO) was reported from Lagos, Nigeria on Jan 31, 2007. The case was a 22 year-old woman whose illness began on January 8^th^, who died on January 16^th^, 2007, and whose mother died on January 4^th^ following a similar illness. The illness in the case-patient’s mother could not be confirmed as AI, since no specimens were collected [7]. Both women had participated in de-feathering a chicken bought from a Lagos chicken market December 21, 2006. (I. Dalhatu, unpub.data) This case was investigated jointly by the Federal and State Ministries of Health and Agriculture, in collaboration with World Health Organization (WHO), the US Centers for Disease Control (CDC), and the Food and Agriculture Organization (FAO). It is the only confirmed human case in WHO-AFRO to date.

Physician reporting is the mainstay of passive outbreak surveillance systems such as the Nigerian Integrated Disease Surveillance and Response system (IDSR). Although the Nigerian government invested in training, awareness, and response activities on physicians’ disease reporting, no study to date has been published investigating issues impacting physician knowledge, perceptions, and practices related to notifiable disease reporting, particularly in the context of human infection with avian influenza. Further, few data describing physician knowledge, attitudes and practices (KAP) for reporting notifiable diseases are available worldwide. In order for public health authorities to respond quickly to potential outbreaks of a new or emerging threat, it is important that health information be distributed to the medical community in a quick and efficient manner. However, little is known regarding how physicians in lower-resourced settings obtain health information, particularly for a new or emerging threat, or their preferred methods of acquiring new health information.

The detection of HPAI in Nigerian poultry, along with continued global circulation of HPAI [8] and the threat of novel avian influenza viruses such as influenza A (H7N9), require physicians to be knowledgeable of the clinical spectrum of human cases of AI and the appropriate mechanisms of reporting these cases to the IDSR, so that these infections do not go undetected.

For this reason, this study was performed to describe physicians’ knowledge, perceptions, and practices related to notifiable disease reporting, their preferred sources for acquiring new health knowledge, and their knowledge level of human avian influenza infection in Nigeria. These findings have implications for reportable disease programs in Africa and beyond.

## Methods

### Study design and setting

Public sector physicians represent the backbone of the Nigerian health care system and therefore are the frontline for reporting of notifiable diseases. For this study, 245 public sector physicians from six of the nine largest Nigerian cities were surveyed during November and December 2008. To obtain geographic representation, the most populous city was selected from each of the 6 geopolitical zones, excluding Lagos and Port Harcourt. Port Harcourt was excluded due to security issues that prevented study activities, and Lagos was excluded due to extensive avian influenza training following the human AI case in 2007. The six selected cities (each with >750,000 population) represent 6.5% of the total population of Nigeria. For inclusion in the study, physicians must have obtained a Bachelor of Medicine, Bachelor of Surgery (M.B.B.S.) or equivalent degree and worked in public hospitals in types of practice that had opportunity to see infectious disease cases, specifically: general practice, family practice, pediatrics, internal medicine, public health, or community medicine. Physicians were excluded if they did not perform clinical work, if they worked exclusively in military hospitals, or if their primary field was surgery or obstetrics/gynecology (OB/GYN).

A single sampling frame was constructed of 3015 eligible physicians identified from rosters of all public hospitals in each of the selected cities (ordered by city, then facility). Use of a single sampling frame provided for inherent weighting of cities and facilities with larger numbers of eligible physicians. From this sampling frame, a cohort of 335 physicians was systematically selected to participate (equal-probability sampling interval of 9), with the assumption of a non-response or refusal rate of 20%. If a sampled physician at a given hospital was not available or not reachable after repeated attempts at contact, the study coordinator selected an alternate physician to interview at random from the same hospital.

### Data collection and analysis

Selected physicians were informed about the study by official correspondence from FMOH and their hospital director, and contacted by telephone to request participation and to schedule the interview. A maximum of eight attempts was made to contact each physician. Each respondent was interviewed by a pair of trained interviewers. A total of 15 interviewers traveled throughout the six geopolitical zones to conduct the interviews, which typically took place in the physician’s office. The survey tool (Additional file 1) was developed by Social and Scientific Systems, Inc. in collaboration with epidemiologists at US CDC and the Nigeria Ministry of Health. It was designed as a hypothesis-generating tool consisting of mostly open-ended questions, where respondents could provide more than one answer to a question. The tool was validated through an extensive interviewer training process with mock interview, mock interview timing, and pilot testing during fall 2008 among physicians at one hospital. Survey components included basic respondent demographics, knowledge of avian influenza, knowledge of notifiable disease reporting procedures and practices related to reporting of avian influenza as well as other epidemic and notifiable diseases, perceived obstacles to reporting, and information access and preferred sources used by the physicians. The interview took approximately 25 minutes to complete.

Dual data entry into a secure data network was performed, and personal identifying information was destroyed once data entry was complete. Data were analyzed using SAS 9.1 statistical software (SAS Institute, Cary, North Carolina). Bivariate analyses were conducted to investigate associations between dependent variables of AI knowledge, information access, notifiable disease reporting practices, and perceived obstacles to reporting and the following independent variables: hospital type, years practicing, type of practice, location of medical training, and amount of clinical supervision received. Categorical data analyses were performed by Pearson’s Chi-squared tests where all expected cell counts were ≥5 and by Fisher’s exact test otherwise. Unless otherwise indicated, p-values are derived from Chi-squared tests. Due to the large number (>100) of one-way comparisons, a conservative p-value of <0.01 was used to define statistical significance. Multivariate logistic regression was performed on the following key outcome variables: correct identification of three key notifiable diseases (avian influenza, polio, and measles); looking up medical information on the internet at least weekly; internet, medical journal, and cell phone text message as a best way to get health information; having seen a notifiable disease; among those seeing notifiable diseases, having reported a notifiable disease, and report of any perceived obstacles to notifiable disease reporting in each of the three main categories (knowledge/attitudes, time/resources, and infrastructure). The following independent variables were included in all models: clinical capacity, years of experience, and hospital type. For each key outcome (x), we used a simple linear regression:

$$ g(x) = {\beta}_0+{\beta}_1\left( years\  of\  experience\right)+{\beta}_2\left( hospital\  type\right)+{\beta}_3\left( clinical\  capacity\right) $$

Variable selection was carried out through backwards selection with a significance cutoff of 0.05 for removal from the model.

### Human subjects

The study protocol was reviewed and approved by the Institutional Review Board (IRB) of the Nigeria Institute of Medical Research (NIMR), and all participants provided written informed consent, in compliance with national regulations governing the protection of human subjects.

## Results

### Respondent demographics

Of the 335 selected physicians, 245 (73%) consented to participate in the survey. Their median age was 37 years, with a range from 28 to 62 years; 165 (67%) were male. The median number of years in medical practice was 9 years, with a range from 1 to 35 years. The participants represented both federal (n = 160, 65%) and state hospitals (n = 85, 35%), as well as teaching (n = 170, 69%) and non-teaching hospitals (n = 75, 31%). Cities represented by the participants were Aba (n = 21, 9%), Abuja (n = 34, 14%), Benin City (n = 73, 30%), Ibadan (n = 52, 21%), Kano (n = 41, 17%), and Maiduguri (n = 24, 10%). Two hundred and seventeen participants (89%) received their medical training in Nigeria only, with only 3 physicians (1%) receiving training exclusively outside Nigeria, and 25 (10%) receiving training both outside and in Nigeria. The most common specialties among the physicians were pediatrics, internal medicine, and general practice. Twenty-five hospitals were represented, with a median of 5 respondents per facility (range 1 to 59).

### Notifiable disease reporting

All respondents identified AI as a notifiable disease, and 99% reported that they would report suspected AI in a patient. However, when asked to whom they would first report a suspected human case of AI, there were a wide variety of responses, including hospital authorities (n = 79, 33%), an immediate supervisor or consultant (n = 30, 12%), and the infection control committee (n = 33, 14%). All participants indicated that epidemic infectious notifiable diseases should be reported, but there was also a wide variety of responses for who should be responsible for reporting this type of notifiable disease to government health authorities. Hospital authorities (n = 118, 48%), physicians (n = 84, 34%), and hospital public health department (n = 73, 30%) were most frequently mentioned, but the infectious control committee (n = 61, 25%), infectious disease consultant (n = 44, 18%), and nurses (n = 34, 14%) were also listed.

In response to questions about physicians’ own experience reporting notifiable diseases, two-thirds responded that they had reported a notifiable disease at some time, including both epidemic infectious diseases and other notifiable diseases (Table 1). Almost all physicians (229/245, 93%) correctly identified polio as a notifiable disease, and 174/245 (71%) correctly identified measles as a notifiable disease. Similarly, 71% overall identified all three diseases (AI, polio, and measles) as notifiable, with no differences by clinical capacity or years of experience, Those in pediatric or family medicine were significantly more likely to have reported a notifiable disease (86% and 75%, respectively) than those in other types of practice (general, internal, public health, emergency medicine) (range 48-60%), p < 0.01. Polio was the notifiable disease most commonly reported by physicians. Among physicians who had seen at least one case of a notifiable disease, 163 indicated that they had reported through IDSR and 19 had not. Among those who had never reported a notifiable disease, the primary reason for not reporting was never having seen a case of a notifiable disease (51/78, 65%), followed by a reported lack of infrastructure/reporting system (8/78, 10%) and not knowing how/to whom to report this information (6/78, 8%).

**Table 1:** **Nigerian physicians**’ **knowledge and practices related to notifiable disease reporting** (**N** = **245**)

**Knowledge of notifiable diseases:**	**Total**	≤**10 years clinical experience** **(n = 145)**	**>** **10 years clinical experience** **(n = 100)**	**p** **-value**
Correctly identified avian influenza (AI)	245 (100%)	145 (100%)	100 (100%)	1.00^†^
Correctly identified polio/acute flaccid paralysis	229 (93%)	134 (92%)	95 (95%)	0.59*
Correctly identified measles	174 (71%)	99 (68%)	75 (75%)	0.32*
Correctly identified all three diseases (AI, polio, and measles)	173 (71%)	99 (68%)	74 (74%)	0.39*
Experience reporting notifiable diseases:				
Have reported a notifiable disease	163 (67%)	94 (64%)	69 (69%)	0.59**
Have never reported a notifiable disease	81 (33%)	50 (34%)	31 (31%)	0.59**
Unsure	1 (0%)	1 (1%)	0 (0%)	0.59**
Reason(s) for never reporting a notifiable disease (n = 78^††^):				
Have never seen a case of notifiable infectious disease	51 (65%)	30/47 (64%)	21/31 (68%)	0.72*
Lack of infrastructure/reporting system	8 (10%)	4 (9%)	4 (13%)	0.71^†^
Don’t know how/to whom to report this information	6 (8%)	5 (11%)	1 (3%)	0.39^†^
Don’t believe that reporting will lead to any government response	5 (6%)	4 (9%)	1 (3%)	0.64^†^
Don’t believe it is their job/thought someone else was responsible	5 (6%)	2 (4%)	3 (10%)	0.38^†^
To protect patient confidentiality	1 (1%)	1 (2%)	0 (0%)	1.00^†^
Too busy to report	1 (1%)	1 (2%)	0 (0%)	1.00^†^
Did not have the appropriate materials (forms, telephone, etc.)	1 (1%)	1 (2%)	0 (0%)	1.00^†^
Other reason (e.g., disease already known in community)	13 (17%)	8 (17%)	5 (16%)	0.93*

*Outcomes were compared by years of experience through Chi-squared test.

^†^Outcomes were compared by years of experience through Fishers exact test.

**P-value for "experience reporting notifiable disease" applies to the overall distribution of responses across all three categories.

^††^Three physicians declined to provide reasons for not reporting.

### Obstacles to reporting

Obstacles to disease reporting were noted by nearly all physicians, with 93% reporting at least one obstacle (Table 2). Obstacles fell primarily into three categories: physician knowledge/perceptions, physician time/resources, and reporting system infrastructure, with a number of reported obstacles in all three categories. Physicians in general practice were less likely to report knowledge-related obstacles than physicians in other capacities (33% vs. 57% overall, p < 0.01), although years of experience was not associated with types of obstacles reported. Among obstacles related physician knowledge/perceptions, primary responses included physicians not knowing that they should report/not knowing which infectious diseases to report (72/227, 29%) and physicians not knowing how or to whom to report (64/227, 26%). Frequently reported obstacles related to time/resources included limited diagnostic/laboratory capacity (54/227, 22%) and that doctors are too busy to report (51/227, 51%). Reported obstacles related to infrastructure included a general lack of infrastructure/logistics or reporting system (76/227, 31%) and the belief that reporting will not lead to any government response (47/227, 19%).

**Table 2:** **Physicians**’ **perceived obstacles to infectious disease reporting** (**N** = **245**)

	**Total**	≤**10 years clinical experience** **(n = 145)**	**>** **10 years clinical experience** **(n = 100)**	**p** **-value**
**Physicians reporting at least 1 obstacle**	227 (93%)	138 (95%)	89 (89%)	0.07*
**Obstacles related to physician knowledge and attitudes**				
Physicians reporting at least 1 obstacle related to physician knowledge/attitudes	140 (57%)	85 (59%)	55 (55%)	0.57*
Doctors do not know that they should report	72 (29%)	38 (26%)	34 (34%)	0.24*
Doctors do not know how or to whom to report	64 (26%)	40 (28%)	24 (24%)	0.69*
Doctors don’t know which diseases to report	24 (10%)	17 (12%)	7 (7%)	0.41*
Doctors may not feel it is important	23 (9%)	16 (11%)	7 (7%)	0.48*
Doctors do not believe it is their job to report	9 (4%)	5 (3%)	4 (4%)	0.78^†^
Doctor may never have seen a disease he/she was required to report	9 (4%)	6 (4%)	3 (3%)	0.74^†^
Doctors may want to protect patient confidentiality	8 (3%)	5 (3%)	3 (3%)	0.79^†^
Other obstacles related to physician knowledge/attitudes (e.g., wrong diagnosis)	10 (4%)	6 (4%)	4 (4%)	0.96^†^
**Obstacles related to physician time and resources**				
Physicians reporting at least 1 obstacle related to physician time/resources	139 (57%)	91 (63%)	48 (48%)	0.02*
Limited diagnostic or laboratory capacity	54 (22%)	37 (26%)	17 (17%)	0.26*
Doctors are too busy to report	51 (21%)	33 (23%)	18 (18%)	0.57*
Doctors feel the reporting process is too complicated or cumbersome	47 (19%)	30 (21%)	17 (17%)	0.65*
Doctors may not have appropriate materials (forms, telephone, etc.)	36 (15%)	19 (13%)	17 (17%)	0.53*
Other time/resources obstacles (e.g.,. lack of manpower, no special remuneration)	8 (3%)	4 (3%)	4 (4%)	0.72^†^
**Obstacles related to reporting system infrastructure**				
Physicians reporting at least 1 obstacle related to reporting system infrastructure	119 (49%)	72 (50%)	47 (47%)	0.68*
Lack of infrastructure/logistics or reporting system	76 (31%)	44 (30%)	32 (32%)	0.75*
Doctors do not believe reporting will not lead to any government response	47 (19%)	30 (21%)	17 (17%)	0.65*
Doctors may believe hospital management will take no action	16 (7%)	11 (8%)	5 (5%)	0.60^†^
Other infrastructure obstacles (e.g., lack of feedback)	25 (10%)	18 (12%)	7 (7%)	0.25*
**Physicians reporting other obstacles**	9 (4%)	7 (5%)	2 (2%)	0.32^†^
**Physicians reporting no obstacles**	18 (7%)	7 (5%)	11 (11%)	0.07*

*Outcomes were compared by years of experience through Chi-squared test.

^†^Outcomes were compared by years of experience through Fishers exact test.

### Public health information sources

Access to information was widespread among the participating physicians (Table 3). Almost all participating physicians (>99%) reported having a cell phone that they currently use, and from which they send text messages at least weekly. Internet use was high overall (86% reported using the internet at least weekly); however, weekly or greater internet use was more common among those with ≤10 years clinical practice (91%, compared to 78% among those with >10 years of practice, Fisher’s p < 0.01). The majority of respondents reported looking up medical information on the internet at least weekly, although this practice was more typical among those with ≤10 years clinical practice (86% vs. 72% among those with >10 years’ experience, p < 0.01).

**Table 3:** **Nigerian physicians**’ **access to health information** (**N** = **245**)

	**Total**	≤**10 years clinical experience** **(n = 145)**	**>** **10 years clinical experience** **(n = 100)**	**p** **-value**
Currently own a cell phone to make/receive calls	244 (99%)	145 (100%)	99 (99%)	0.23^†^
Currently have an email address	231 (94%)	140 (97%)	91 (91%)	0.07*
**Access the following at least weekly:**				
Send text messages on cell phone	243/244 (99%)	144 (99%)	99/99 (100%)	0.67^†^
Watch TV	242 (99%)	143 (99%)	99 (99%)	0.79^†^
Use the internet	210 (86%)	132 (91%)	78 (78%)	<0.01*
Check email	208 (85%)	125 (86%)	83 (83%)	0.49*
Listen to the radio	201 (82%)	118 (81%)	83 (83%)	0.75*
Look up medical information on the internet	197 (80%)	125 (86%)	72 (72%)	<0.01*
**Usual location for internet access:**				
Hospital	106/242 (44%)	69 (48%)	37/97 (38%)	0.23**
Home	65/242 (27%)	41 (28%)	24/97 (25%)	0.23**
Internet café	56/242 (23%)	28 (19%)	28/97 (29%)	0.23**
Other (e.g., wireless)	15/242 (6%)	7 (5%)	8/97 (8%)	0.23**

*Outcomes were compared by years of experience through Chi-squared test.

^†^Outcomes were compared by years of experience through Fishers exact test.

**P-value for "usual location for internet access" applies to the overall distribution of responses across all four categories.

When asked about the best ways to deliver health information to the physicians, medical journals and internet websites were the most common preferred sources, followed by training sessions and email (Table 4). Medical journals were more commonly mentioned among those with >10 years’ experience, compared to those with ≤ 10 years’ experience (60% vs. 39%, p < 0.01). Cell phones were listed by 12/245 (5%) of physicians as a preferred source for general health information. The most useful source for AI-related information reported by physicians was the internet. Least commonly listed for either general health information or AI-related information were pamphlets, Ministry of health communications, posters, billboards, and community enlightenment meetings.

**Table 4:** **Nigerian physicians**’ **preferred sources for general health information and avian influenza** (**AI**) (**N** = **245**)

	Best ways to get health information*				**Most useful source for learning about AI** ^**†**^			
**Source:**	**Total**	≤**10 years clinical experience (n = 145)**	**>10 years clinical experience (n = 100)**	**p-value**	**Total**	≤**10 years clinical experience (n = 145)**	**>10 years clinical experience (n = 100)**	**p-value**
Medical journal	117 (48%)	57 (39%)	60 (60%)	<0.01**	21 (9%)	13 (9%)	8 (8%)	0.97**
Internet website	105 (43%)	64 (44%)	41 (41%)	0.63**	63 (26%)	39 (27%)	24 (24%)	0.13**
#1 internet site	www.google.com	www.google.com	www.google.com		www.google.com	www.google.com	www.google.com	
#2 internet site	www.eMedicine.com	www.eMedicine.com	www.pubmed.com		www.who.int	www.who.int	www.who.int	
#3 internet site	www.who.int	www.who.int	www.who.int		www.cdc.gov	www.cdc.gov	www.cdc.gov	
Training sessions/seminars/workshops	94 (38%)	62 (42%)	32 (32%)	0.09**	33 (13%)	21 (14%)	12 (12%)	0.14**
Email	68 (28%)	34 (23%)	24 (34%)	0.07**	0 (0%)	0 (0%)	0 (0%)	1.00^††^
Medical book	60 (24%)	44 (30%)	16 (16%)	0.01**	20 (8%)	14 (10%)	6 (6%)	0.43**
Television	43 (18%)	27 (19%)	16 (16%)	0.60**	49 (20%)	26 (18%)	23 (23%)	0.66**
Newspapers/magazines	37 (15%)	23 (16%)	14 (14%)	0.69**	14 (6%)	6 (4%)	8 (8%)	0.32**
Radio	27 (11%)	16 (11%)	11 (11%)	0.99**	4 (2%)	2 (1%)	2 (2%)	1.00^††^
Educational lecture/course	26 (11%)	18 (12%)	8 (8%)	0.27**	9 (4%)	8 (6%)	1 (1%)	0.09^††^
Pamphlets	18 (7%)	11 (8%)	7 (7%)	0.86**	3 (1%)	0 (0%)	3 (3%)	0.07^††^
Professional colleagues	14 (6%)	7 (5%)	7 (7%)	0.47**	15 (6%)	10 (7%)	5 (5%)	0.74**
Cell phone text message	12 (5%)	7 (5%)	5 (5%)	0.95**	0 (0%)	0 (0%)	0 (0%)	1.00^††^
Ministry of health communication	10 (4%)	6 (4%)	4 (4%)	1.00^††^	2 (1%)	1 (1%)	1 (1%)	1.00^††^
Medical school/postgraduate training	3 (1%)	2 (1%)	1 (1%)	1.00^††^	0 (0%)	0 (0%)	0 (0%)	1.00^††^
Posters	4 (2%)	2 (1%)	2 (2%)	1.00^††^	1 (<1%)	1 (1%)	0 (0%)	1.00^††^
Billboards	1 (<1%)	1 (1%)	0 (0%)	1.00^††^	0 (0%)	0 (0%)	0 (0%)	1.00^††^
Public lecture/community enlightenment	1 (<1%)	1 (1%)	0 (0%)	1.00^††^	0 (0%)	0 (0%)	0 (0%)	1.00^††^
Other	50 (20%)^‡^	27 (19%)	23 (23%)	0.40**	9 (4%)	4 (3%)	5 (5%)	0.49**

*Multiple responses accepted.

^†^Single response accepted.

**Outcomes were compared by years of experience through Chi-squared test.

^††^Outcomes were compared by years of experience through Fishers exact test.

^‡^“Other” best ways to get health information included CD-ROM, hard copy, and mail.

### Knowledge of avian influenza

All 245 respondents reported that they had heard of AI (also referred to “bird flu”), with 171 (70%) rating their knowledge as “average”, 23 (9%) as “very knowledgeable” and 51 (21%) as “not very knowledgeable”. All 245 (100%) also reported that humans could get AI, and a majority (n = 177, 72%) correctly identified common signs and symptoms of AI infection in humans, although fewer identified gastrointestinal symptoms such as diarrhea and vomiting (n = 62, 25%), which are listed by WHO as early symptoms of AI infection. A large minority (n = 67, 28%) incorrectly indicated rash as a sign/symptom of AI infection. When asked about how AI could be transmitted, most respondents correctly identified all of the following modes of transmission: contact with sick/dead chickens (n = 232, 95%), sick/dead wild birds (n = 218, 89%), droppings from sick/dead chickens (n = 231, 94%), raw/undercooked meat from sick/dead chickens (n = 236, 97%), or contact with humans infected with AI (n = 230, 94%). Nearly all respondents also correctly indicated that mosquito bites (n = 236, 96%) and properly cooked poultry (n = 230, 94%) were not modes of AI transmission in humans.

Logistic regression analyses found no significant multivariate associations with any of the outcomes of interest. For each multivariate regression, a maximum of one independent variable remained in the model after variable selection and convergence. Additionally, all multivariate regressions showed a poor model fit, with likelihood-based pseudo R-square values ranging from 0.01 to 0.05, with most falling near 0.02.

## Discussion

In order to effectively identify human infections with avian influenza and other emerging diseases through the current IDSR system, reporting system requirements need to be clearly communicated to the participating physicians. Despite widespread awareness of AI including common symptoms and modes of transmission, there was no consensus among participating physicians to whom they should report notifiable diseases including AI, and physicians noted many obstacles to reporting. Primary obstacles reported included a lack of reporting infrastructure; doctors not knowing how or to whom they should report within their facility or district, or that they should report at all; and limited diagnostic capabilities. Physicians in general practice were less likely to report knowledge-related obstacles than physicians in other clinical capacities, such as pediatrics or internal medicine. Improved laboratory capacity for diagnostic confirmation could further strengthen the IDSR system, as it could serve as an alternative mechanism for reporting cases to local, state, and federal health authorities. However, this would still rely on physicians’ identification of cases for diagnostic testing.

Physicians reported widespread access to information, identifying methods such as internet, cell phone, and email that could be better utilized to reach this population for regular updates and for crisis communications, such as epidemic or pandemic activity. Despite some variation in internet usage by years of experience (both in frequency of general internet use and in looking up medical information), internet sites were considered by respondents of all levels of experience to be the best source of information about avian influenza, which may serve as an indicator of best source of information for other emerging disease threats. The World Health Organization implemented this strategy during the 2009 influenza A (H1N1) pandemic, improving collection and dissemination of information at the global level [9]. Although cell phones were not frequently cited as a best way to get health information, their ubiquity among physicians may provide a method of reaching almost all of this population quickly and cheaply. Cell phone short message services (SMS)/text messages have been identified as a key surveillance system enhancement for improving pandemic preparedness in African settings [10]. Additionally, educational lectures/courses, as well as pamphlets, posters, and other methods of communication commonly used to inform this audience, were listed very infrequently as a best way of communicating health information or as a most useful source of AI, and may not be as effective for future awareness campaigns.

Timely dissemination of information such as clinical presentations and reporting procedures is an integral part of public health surveillance and outbreak response. A flexible, cost-efficient and rapid method for spreading information to physicians and public health officials is vital for the success of these event-reporting systems, especially in countries with limited resources. Electronic methods such as the internet, email distribution lists and use of cell phones have significant potential in this area and the results of this study indicate that physicians reported them to be at least as useful as traditional methods as ways to obtain health information and most useful in for learning about a newly emerging disease (AI). Since these methods can be more flexible, faster, and less expensive to use than traditional trainings, workshops, lectures and publications in traditional scientific media, further exploration of the utilization of these electronic methods for information distribution should be investigated.

These findings are consistent with previous surveys of Nigerian health care workers’ awareness of the IDSR system [11-13]. Although physician training has demonstrated a positive impact on IDSR and notifiable disease reporting [12], there remain low levels of awareness of the IDSR system and a continued need for infrastructure improvements such as more frequent data analysis and increased feedback to health facilities [13]. These findings also expand the global understanding of physicians’ knowledge, attitudes, and practices for notifiable disease reporting, which has been predominated to date by surveys of physicians in higher income settings in the US, Canada, and Europe [14-18]. Physicians in our study reported less frequently than in Friedman *et al*. [14] and Konowitz *et al*. [15] that knowing which diseases were notifiable was an obstacle to reporting, despite the larger number of notifiable diseases in this population (n = 23) versus those in the other two study populations (n = 16 and n = 20, respectively). This difference may reflect either widespread awareness among our study population of which diseases are notifiable or a reluctance to report a potential lack of awareness. However, there were many similarities to surveys in Germany and the US, which identified a lack of feedback to physicians as a key obstacle to the reporting system [16] and a lack of awareness regarding notifiable disease reporting procedures [15,18]. The lack of published data from neighboring African countries limits regional comparison of the current findings.

Overall, knowledge of notifiable epidemic infectious diseases (including AI, polio, and measles) was relatively high, with no trends in knowledge by clinical capacity or years of experience, although there remains some room for improvement as almost one-third of physicians did not correctly identify all three as being notifiable diseases. Knowledge of AI was used in this study as a proxy for how well physicians in Nigeria acquire knowledge of emerging or new health threats. Following the trainings and media campaign following the outbreak of HPAI H5N1 in Nigeria in the 2 years prior to the study, this study showed that information regarding this emerging disease had penetrated all areas under study. Since over 20% of physicians were not able to identify the common signs and symptoms of AI infection in humans, continued evaluation and modification of training and outreach campaigns for notifiable and emerging diseases should be performed.

This study was subject to several limitations. The participants were all from public hospitals in urban centers, and thus the findings presented here may not represent knowledge, perceptions and practices among rural, military or private facilities. Of note, the FMOH installed surveillance officers in public facilities to facilitate reporting, so private clinics without surveillance officers may have more limited infrastructure and lower levels of reporting. Regression analysis did not identify any significant multivariate predictors of key study outcomes such as identifying or reporting notifiable diseases; however, there may have been additional predictors of these outcomes that were not captured by our questionnaire. An effort was made to obtain geographic representation by selecting the most populous city from each of the six geopolitical zones; however, cities from two zones were excluded. Lagos was excluded because this region was specifically targeted for increased avian influenza and IDSR training following the human AI case in 2007 and this might have biased the study results. Port Harcourt was excluded due to security issues that prevented study activities. These security issues may have had an impact on the reporting system infrastructure and therefore the results may not be generalizable to physicians from this area.

## Conclusions

Although the majority of physicians surveyed, regardless of clinical capacity, hospital type, or years of experience, were knowledgeable of notifiable diseases and had reported notifiable diseases, they identified many perceived obstacles to notifiable disease reporting. In order to effectively identify AI and other infectious diseases through IDSR, reporting system requirements need to be clearly communicated to the participating physicians and perceived obstacles, such as lack of infrastructure, addressed. Future improvements to the reporting system targeting this population could benefit from increased utilization of the internet, as well as SMS and email-based communication.

## Additional file

Additional file 1:
**Avian influenza survey.**
