# Supplementary material for: Notifiable disease reporting among public sector physicians in Nigeria: a cross-sectional survey to evaluate possible barriers and identify best sources of information
Source: BMC Health Serv Res. 2014 Nov 13;14:568. doi: 10.1186/s12913-014-0568-3 (PMC4233090; doi:10.1186/s12913-014-0568-3)
Supplement: Additional file 1: — Avian influenza survey. [file 12913_2014_568_MOESM1_ESM.doc]

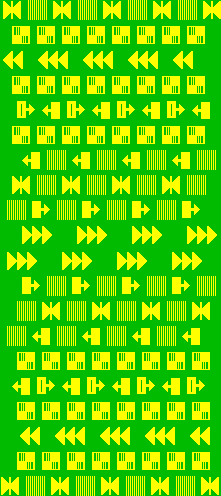

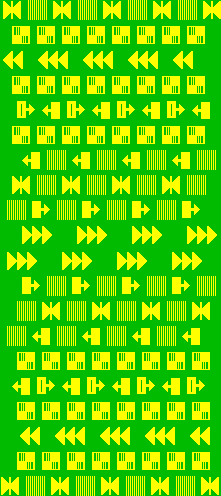

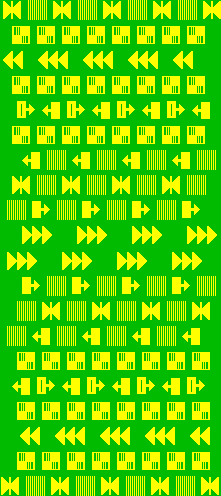

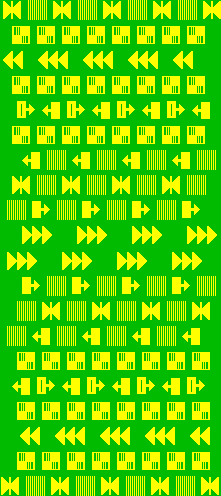

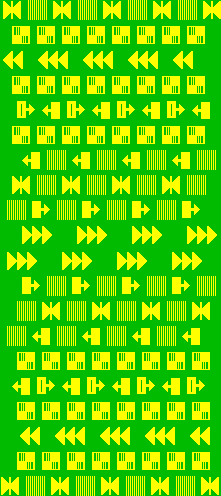

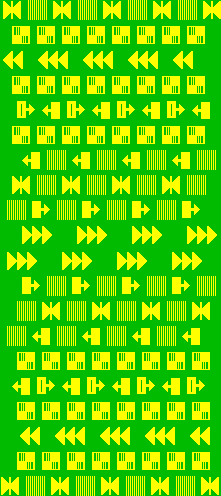

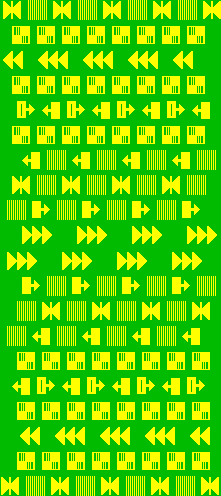


**1 Avian Influenza Survey 1**

Survey of Knowledge, Attitudes, and Practices Related to Reporting of Human Infections of Avian Influenza and other Notifiable Diseases by Physicians and Nurses in Nigeria

DAY MONTH YEAR

**/**

**/**

**Interviewer Initials**: **Today’s Date**:

(Date)

(Int)

**City**: ❑ 1 Aba ❑ 2 Abuja ❑ 3 Benin City **Hospital Identification**:

(FacID)

(City)

❑ 4 Ibadan ❑ 5 Kano ❑ 6 Maiduguri

**Type of Government Hospital:** ❑ 1 Federal **Type of Hospital:** ❑ 1 Teaching Hospital

(HospType)

(GovHosp)

❑ 2 State ❑ 2 Non-teaching Hospital

**Respondent’s Gender**: ❑ 1 Female

❑ 2 Male

(ResGen)

**IMPORTANT INSTRUCTIONS FOR INTERVIEWER**

- Unless otherwise indicated, DO NOT READ RESPONSE OPTIONS.
- Unless otherwise noted, more than one answer per question is possible. MARK ALL THAT APPLY.
- Follow the instructions carefully for each item as they vary across questions.

We are interested in learning what doctors know, think, and do when faced with avian influenza (also called bird flu) and other reportable, infectious diseases. We also want to learn what might stop someone from reporting a suspected or confirmed case of a reportable, infectious disease. And, finally, we would like to understand how doctors in Nigeria get medical and public health information.

I am going to ask you several questions. Please understand that there are no right or wrong answers. Just answer the questions to the best of your ability. The first few questions are about your knowledge of avian influenza, also called bird flu.

Form: 1 Version: 1 Revision Date 08/22/2008

**Avian Influenza Survey 2**

**SECTION A: Avian Influenza Knowledge**

**AF1.** Have you heard of avian influenza or bird flu?

(AF1)

❑ 1 YES

❑ 2 NO

**IF “NO”, “REFUSED”, or “DON’T KNOW”**

**GO TO SECTION C, QUESTION AF16 ON PAGE 8**

❑ 7 REFUSED

❑ 8 DON’T KNOW

1 = box marked

2 = no mark in box

7 = “97” marked (Refused)

8 = “98” marked (Don’t Know)

**AF2** From what sources did you learn information about bird flu? **[DO NOT READ OUT RESPONSE OPTIONS] [AFTER EACH RESPONSE, PROBE: “Anywhere else?”]**

(AF2_1) ❑ 1 Educational lecture or course

(AF2_2) ❑ 2 Training sessions/seminars/workshops

(AF2_3) ❑ 3 State or federal ministry of health communication (for example: letter, bulletin, or newsletter)

(AF2_4) ❑ 4 Medical book

(AF2_5) ❑ 5 Medical journal

(AF2_6) ❑ 6 Medical school/or postgraduate medical training (for example: internship, residency, or fellowship)

(AF2_7) ❑ 7 Professional colleagues

(AF2_8) ❑ 8 Pamphlets

(AF2_9) ❑ 9 Posters

(AF2_10) ❑ 10 Billboards

(AF2_11) ❑ 11 Internet website

**AF2web**. Specify website: _______________________________________________

(AF2_12) ❑ 12 Email

(AF2_13) ❑ 13 Newspaper/magazines

(AF2_14) ❑ 14 Radio

(AF2_15) ❑ 15 Television

(AF2_16) ❑ 16 Cell phone text message or short message service (SMS)

(AF2_17) ❑ 17 Public lecture or through community enlightenment

(AF2_18) ❑ 18 Neighbors/friends

(AF2_19) ❑ 19 Religious or traditional leader (tribal, village, or community leader)

(AF2_20) ❑ 20 Other source

**AF2sp1.** Specify other source: _______________________________________________

**AF2sp2.** Specify other source: _______________________________________________

**AF2sp3**. Specify other source: _______________________________________________

❑ 97 REFUSED

**IF “REFUSED” or “DON’T KNOW” GO TO QUESTION AF4.**

❑ 98 DON’T KNOW

**3 Avian Influenza Survey**

**AF3.** Of the sources you just mentioned, which one source provided the most useful information?

**[WRITE OUT NUMBER FOR THE APPROPRIATE RESPONSE]**

**[ONLY ONE ANSWER IS POSSIBLE]**

❑ 97 REFUSED

❑ 98 DON’T KNOW

**AF4.** How would you rate your knowledge of bird flu**? [READ OUT RESPONSE OPTIONS]**

**[ONLY ONE ANSWER IS POSSIBLE]**

❑ 1 Very knowledgeable

❑ 2 Average

❑ 3 Not very knowledgeable

❑ 4 No knowledge

❑ 7 REFUSED

❑ 8 DON’T KNOW

**AF5.** Can humans get bird flu?

❑ 1 YES

❑ 2 NO

**IF “NO”, “REFUSED”, or “DON’T KNOW”**

**GO TO SECTION C, QUESTION AF16 ON PAGE 8**

❑ 7 REFUSED

❑ 8 DON’T KNOW

**AF6**. Now I will ask about ways people can get bird flu.

**For each of the following questions please answer: “yes” or “no.”**

| **[READ EACH QUESTION]**  (AF6A)… | **Yes** | **No** | **REFUSED** | **DON’T KNOW** |
| --- | --- | --- | --- | --- |
| **AF6a**. From touching or handling sick or dead chickens? | ❑ 1 | ❑ 2 | ❑ 7 | ❑ 8 |
| **AF6b.** From eating properly cooked chickens or eggs? | ❑ 1 | ❑ 2 | ❑ 7 | ❑ 8 |
| **AF6c.** From eating raw or undercooked meat from sick  (AF6C) or dead chickens? | ❑ 1 | ❑ 2 | ❑ 7 | ❑ 8 |
| **AF6d.** From touching surfaces contaminated with (AF6D) droppings from sick or dead chickens? | ❑ 1 | ❑ 2 | ❑ 7 | ❑ 8 |
| **AF6e**. From touching sick or dead horses or cattle? | ❑ 1 | ❑ 2 | ❑ 7 | ❑ 8 |
| **AF6f.** From providing healthcare to people with bird flu (AF6F) without wearing personal protective equipment? | ❑ 1 | ❑ 2 | ❑ 7 | ❑ 8 |
| **AF6g.** From mosquito bites? | ❑ 1 | ❑ 2 | ❑ 7 | ❑ 8 |
| **AF6h.** From eating or touching sick or dead wild birds?  (AF6H) [including handling, slaughtering, defeathering, or butchering] | ❑ 1 | ❑ 2 | ❑ 7 | ❑ 8 |

**Avian Influenza Survey 4**

**AF7.** If a person is sick with bird flu, how likely are they to die from bird flu?

**[READ OUT RESPONSE OPTIONS] [ONLY ONE ANSWER IS POSSIBLE]**

❑ 1 Unlikely

❑ 2 Likely

❑ 3 Very likely

❑ 7 REFUSED

❑ 8 Don’t know

**AF8.** What age group is at most risk for **getting** bird flu?

**[READ OUT RESPONSE OPTIONS] [ONLY ONE ANSWER IS POSSIBLE]**

❑ 1 Children less than 5 years old

❑ 2 Children 5 to 17 years old

❑ 3 Adults age 18 to 60

❑ 4 Adults over age 60

❑ 7 REFUSED

❑ 8 Don’t know

**AF9**. Which of the following signs and symptoms are seen in patients with bird flu infection?

For each sign or symptom, answer “yes” or “no.”

|  | **YES** | **NO** | **REFUSED** | **DON’T KNOW** |
| --- | --- | --- | --- | --- |
| **AF9a.** High fever | ❑ 1 | ❑ 2 | ❑ 7 | ❑ 8 |
| **AF9b.** Sore throat | ❑ 1 | ❑ 2 | ❑ 7 | ❑ 8 |
| **AF9c.** Cough | ❑ 1 | ❑ 2 | ❑ 7 | ❑ 8 |
| **AF9d.** Difficulty breathing or shortness of breath | ❑ 1 | ❑ 2 | ❑ 7 | ❑ 8 |
| **AF9e.** Runny nose | ❑ 1 | ❑ 2 | ❑ 7 | ❑ 8 |
| **AF9f.** Sore eyes/ conjunctivitis | ❑ 1 | ❑ 2 | ❑ 7 | ❑ 8 |
| **AF9g.** Rash | ❑ 1 | ❑ 2 | ❑ 7 | ❑ 8 |
| **AF9h.** Muscle aches or myalgia | ❑ 1 | ❑ 2 | ❑ 7 | ❑ 8 |
| **AF9i.** Diarrhea | ❑ 1 | ❑ 2 | ❑ 7 | ❑ 8 |
| **AF9j.** Vomiting | ❑ 1 | ❑ 2 | ❑ 7 | ❑ 8 |
| **AF9k.** Headache | ❑ 1 | ❑ 2 | ❑ 7 | ❑ 8 |

**5 Avian Influenza Survey**

**AF10.** In which of the following situations would you suspect bird flu in a patient? For each situation, answer “**would suspect**” or “**would not suspect**” bird flu.

|  | **Would suspect** | **Would not**  **suspect** | **REFUSED** | **DON’T KNOW** |
| --- | --- | --- | --- | --- |
| **AF10a.** If the patient has eaten properly cooked (AF10A) eggs and then develops shortness of breath | ❑ 1 | ❑ 2 | ❑ 7 | ❑ 8 |
| **AF10b.** If the patient has been taking care of another suspected bird flu patient and develops fever, sore throat, and shortness of breath | ❑ 1 | ❑ 2 | ❑ 7 | ❑ 8 |
| **AF10c.** If the patient has eaten sick chicken and then develops fever, cough, and shortness of breath | ❑ 1 | ❑ 2 | ❑ 7 | ❑ 8 |
| **AF10d.** If the patient has handled dead chickens and has developed only a fever | ❑ 1 | ❑ 2 | ❑ 7 | ❑ 8 |
| **AF10e.** If the patient has butchered healthy chickens and then develops fever, cough, and shortness of breath | ❑ 1 | ❑ 2 | ❑ 7 | ❑ 8 |

**AF11.** If you had a patient with bird flu, what **specific** precautions would you take to protect yourself and others from becoming infected? **[DO NOT READ OUT RESPONSE OPTIONS]**

**[PROBE WITH EACH RESPONSE: “Any other ways?”]**

AF11_1❑ 1 Wear gloves

1 = box marked

2 = no mark in box

7 = “97” marked (Refused)

8 = “98” marked (Don’t Know)

AF11_2❑ 2 Wear a mask

AF11_3❑ 3 Wear a respirator

AF11_4❑ 4 Wear a gown

AF11_5❑ 5 Wear goggles or other eye protection

AF11_6❑ 6 Wash your hands after seeing the patient

AF11_7❑ 7 Minimize contact with the patient

AF11_8❑ 8 Put the patient in an isolation room or a room for a single patient

AF11_9❑ 9 Have the patient wear a mask

AF11_10❑ 10 Refuse to see the patient

AF11_11❑ 11 Other

**AF11sp1.** Specify_________________________________________________________

**AF11sp2.** Specify_________________________________________________________

**AF11sp3.** Specify_________________________________________________________

❑ 97 REFUSED

❑ 98 DON’T KNOW

**Avian Influenza Survey 6**

**SECTION B: Reporting Avian Influenza**

Next, I would like to ask you about reporting patients infected with bird flu.

**AF12.** If you suspect your patient has bird flu, would you report this patient’s illness?

❑ 1 YES

**IF “NO”, “REFUSED”, or “DON’T KNOW”,**

**GO TO SECTION C, QUESTION AF16 ON PAGE 8.**

❑ 2 NO

❑ 7 REFUSED

❑ 8 DON’T KNOW

**AF13.** To whom would you **first** report your patient’s illness?

**[DO NOT READ OUT RESPONSE OPTIONS]**

(AF13) **[DO NOT PROBE AFTER RESPONSE] [ONLY ONE ANSWER IS POSSIBLE]**

❑ 1 Hospital authorities [Head of Department (HOD), Chief Medical Director (CMD), or hospital administrative leaders]

❑ 2 Immediate supervisor/consultant

❑ 3 Infectious disease consultant

❑ 4 Hospital’s public health/community health department

❑ 5 Infection control committee

❑ 6 Medical record officer (MRO)

❑ 7 Local government DSNO or PHC personnel

❑ 8 Other state officials (not including DSNO or PHC personnel)

❑ 9 State Ministry of Health

❑ 10 Federal Ministry of Health

❑ 11 Local veterinarian

16 = Other (specified in AF13sp)

16 = More than one answer marked

(numeric code of response recorded in AF13sp)

❑ 12 Avian influenza hotline

❑ 13 Television or radio station

❑ 14 Newspaper

❑ 15 Religious or traditional leader (tribal, village, or community leader)

❑ 16 Other

**AF13sp**. Specify: _____________________________________________________________

❑ 97 REFUSED

❑ 98 DON’T KNOW

**AF14.** To whom else would you report your patient’s illness?

**[DO NOT READ OUT RESPONSE OPTIONS]**

**[PROBE WITH EACH RESPONSE: “Anyone else?”]**

AF14_1❑ 1 Hospital authorities [Head of Department (HOD), Chief Medical Director (CMD), or hospital administrative leaders]

AF14_2❑ 2 Immediate supervisor/consultant

1 = box marked

2 = no mark in box

7 = “97” marked (Refused)

8 = “98” marked (Don’t Know)

AF14_3❑ 3 Infectious disease consultant

AF14_4❑ 4 Hospital’s public health/community health department

AF14_5❑ 5 Infection control committee

AF14_6❑ 6 Medical record officer (MRO)

AF14_7❑ 7 Local government DSNO or PHC personnel

AF14_8❑ 8 Other state officials (not including DSNO or PHC personnel)

AF14_9❑ 9 State ministry of health

AF14_10❑ 10 Federal ministry of health

AF14_11❑ 11 Local veterinarian

AF14_12❑ 12 Avian influenza hotline

AF14_13❑ 13 Television or radio station

AF14_14❑ 14 Newspaper

AF14_15❑ 15 Religious or traditional leader (tribal, village, or community leader)

AF14_16❑ 16 Other

**AF14sp1**  Specify _______________________________________________________________

16 = Other (specified in AF14sp1)

16 = No one else (specified in AF14sp1)

**AF14sp2**  Specify _______________________________________________________________

**AF14sp3.**  Specify _______________________________________________________________

❑ 97 REFUSED

❑ 98 DON’T KNOW

**AF15.** Within what period of time would you report a patient with **suspected** bird flu?

**7 Avian Influenza Survey**

**[READ RESPONSE OPTIONS] [ONLY ONE ANSWER IS POSSIBLE]**

❑ 1 immediately (less than half a day)

❑ 2 within 1 day

❑ 3 within 2 to 6 days

❑ 4 within 1 to 4 weeks

❑ 5 more than one month

❑ 6 would wait until laboratory confirmation

❑ 7 REFUSED

❑ 8 Don’t know

**Avian Influenza Survey 8**

**SECTION C: Reporting Infectious Diseases**

The following set of questions is about reporting infectious diseases, in general, not just bird flu.

Remember there are no right or wrong answers.

**AF16.** A notifiable infectious disease is one that requires reporting to government health authorities when the diagnosis is suspected. In the past, have you **ever** reported a case of a notifiable infectious disease to your hospital’s authorities or to government health authorities?

❑ 1 YES

**IF AF16 = “NO”, GO TO QUESTION AF18 ON PAGE 9.**

❑ 2 NO

❑ 7 REFUSED

**IF AF16 = “REFUSED”, or “DON’T KNOW”**

**GO TO QUESTION AF20 ON PAGE 10.**

❑ 8 DON’T KNOW

**AF17.** What NOTIFIABLE INFECTIOUS diseases have you reported to hospital authorities or to government health authorities? **[DO NOT READ OUT RESPONSE OPTIONS]**

**[PROBE WITH EACH RESPONSE: “Any others?”]**

AF17_1❑ 1 AIDS

AF17_2❑ 2 Diarrhea

AF17_3❑ 3 Malaria

AF17_4❑ 4 Pneumonia

AF17_5❑ 5 Sexually Transmitted Disease

AF17_6❑ 6 Tuberculosis

AF17_7❑ 7 Polio/ Acute Flaccid Paralysis

AF17_8❑ 8 Dracunculiasis or Guinea Worm

AF17_9❑ 9 Leprosy

AF17_10❑ 10 Neonatal Tetanus

AF17_11❑ 11 Cholera

AF17_12❑ 12 Diarrhea with blood

AF17_13❑ 13 Measles

AF17_14❑ 14 Meningitis

AF17_15❑ 15 Plague

AF17_16❑ 16 Viral Hemorrhagic Fever (example: Lassa Fever)

AF17_17❑ 17 Avian Influenza or bird flu

AF17_18❑ 18 Yellow Fever

AF17_19❑ 19 Other disease

**AF17sp1** Specify:_______________________________________________________

**AF17sp2** Specify:_______________________________________________________

**AF17sp3.** Specify:_______________________________________________________

❑ 97 REFUSED

❑ 98 DON’T KNOW

**FOR ALL RESPONSES TO AF17, GO TO QUESTION AF20 ON PAGE 10.**

**9 Avian Influenza Survey**

**Answer AF18 ONLY IF AF16 = “NO”**

**AF18.** What is the reason you have not reported a notifiable infectious disease?

**[DO NOT READ OUT RESPONSE OPTIONS]**

**[PROBE WITH EACH RESPONSE: “Any other reasons?”]**

AF18_1❑ 1 I have never seen an infectious disease that I was required to report

AF18_2❑ 2 Did not know that I had to report

AF18_3❑ 3 Did not feel it was important to report / I didn’t care to report

AF18_4❑ 4 To protect the confidentiality of the patient with the disease

AF18_5❑ 5 Don’t know which diseases to report

AF18_6❑ 6 Too busy, I don’t have time to report

❑ 7 I don’t believe that reporting will lead to any response by the government authorities

❑ 8 I don’t believe it is my job to report / thought that someone else was responsible for reporting

❑ 9 I don’t know how to or to whom to report this information

❑ 10 Lack of infrastructure or reporting system

❑ 11 I did not have the appropriate materials for reporting (forms, telephone, etc)

❑ 12 Other reason,

**AF18sp1** Specify ________________________________________________________

**AF18sp2** Specify ________________________________________________________

**AF18sp3.** Specify ________________________________________________________

❑ 97 REFUSED

❑ 98 DON’T KNOW

**AsK AF19 IF AT LEAST ONE OF THE RESPONSES IN AF18 = “7.” All OTHERS GO TO AF20 ON PAGE 10**

**AF19**. Why do you believe that reporting a notifiable infectious disease will not lead to any response by the government health authorities?” **[DO NOT READ OUT RESPONSE OPTIONS]**

**[PROBE WITH EACH RESPONSE: “Any other reasons?”]**

AF19_1❑ 1 Lack resources (human or physical ) to respond

AF19_2❑ 2 Lack expertise to respond / don’t know how to respond

AF19_3❑ 3 Health authorities do not want to respond

AF19_4❑ 4 Health authorities are too busy to respond

AF19_5❑ 5 It is not their responsibility to respond

AF19_6❑ 6 Other reason

**AF19sp1** Specify _________________________________________________

**AF19sp2** Specify _________________________________________________

**AF19sp3.** Specify _________________________________________________

❑ 7 REFUSED

❑ 8 DON’T KNOW

**Avian Influenza Survey 10**

**AF20.** These next questions are about **epidemic infectious** **disease** - that is, a communicable disease which is very (AF20) contagious and can cause many people to become ill quickly, for example cholera or Lassa fever. In the past, have you or your hospital reported an **epidemic infectious** **disease** to your local or state government?

❑ 1 YES

❑ 2 NO

**IF “NO”, “REFUSED”, or “DON’T KNOW”**

**GO TO QUESTION AF22 ON PAGE 11**

❑ 7 REFUSED

❑ 8 DON’T KNOW

**AF21.** In the past, when you or your hospital reported an **epidemic infectious disease** to your local or state government, what actions did the government health authorities take?

**[DO NOT READ OUT RESPONSE OPTIONS]**

**[PROBE WITH EACH RESPONSE: “Any other actions?”]**

(AF21_1) ❑ 1 Verified/confirmed case and/or conducted an investigation of the case patient(s)

(AF21_2) ❑ 2 Provided medical supplies / medicines for treatment of patients with the disease

(AF21_3) ❑ 3 Provided financial support

(AF21_4) ❑ 4 Assisted with controlling the spread of the infectious disease in the hospital and or the community/village

(AF21_5) ❑ 5 Alerted the other government authorities about the situation

❑ 6 Alerted tribal, traditional, village, local, community or religious leaders about the problem

❑ 7 Gave advice on the treatment and management of the disease

❑ 8 Provided public enlightenment / education to the public

❑ 9 Provided referral and transport services

❑ 10 Restricted movement

❑ 11 No action was taken

❑ 12 Other action

**AF21sp1** Specify___________________________________________________________

**AF21sp2** Specify___________________________________________________________

**AF21sp3.** Specify___________________________________________________________

❑ 97 REFUSED

❑ 98 Don’t know

**11 Avian Influenza Survey**

**AF22.** When an **epidemic infectious disease** is reported to government health authorities, what actions do you expect the local government health authorities to take?

**[DO NOT READ OUT RESPONSE OPTIONS]**

**[PROBE WITH EACH RESPONSE: “Any other actions?”]**

(AF22_1) ❑ 1 Verify/confirm case and/or conduct an investigation of the case patient(s)

(AF22_2) ❑ 2 Provide medical supplies / medicines for treatment of patients with the disease

(AF22_3) ❑ 3 Provide financial support

(AF22_4) ❑ 4 Assist with controlling the spread of the infectious disease in the hospital and or the community/village

(AF22_5) ❑ 5 Alert the other government authorities about the situation

❑ 6 Alert tribal, traditional, village, local, community or religious leaders about the problem

❑ 7 Give advice on the treatment and management of the disease

❑ 8 Provide public enlightenment / education to the public

❑ 9 Provide referral and transport services

❑ 10 Restrict movement

❑ 11 I don’t expect any action will be taken

❑ 12 Other action

**AF22sp1** Specify____________________________________________________________

**AF22sp2** Specify____________________________________________________________

**AF22sp3.** Specify____________________________________________________________

❑ 97 REFUSED

❑ 98 Don’t know

**ASK AF23 ONLY IF AT LEAST ONE OF THE RESPONSES IN AF22 = “11.” ALL OTHERS GO TO AF24 ON PAGE 12**

**AF23.** Why do you expect that no action would be taken by the government health authorities when an epidemic infectious disease is reported to them?” **[DO NOT READ OUT RESPONSE OPTIONS]**

**[PROBE WITH EACH RESPONSE: “Any other reasons?”]**

AF23_1❑ 1 Lack resources (human or physical ) to respond

AF23_2❑ 2 Lack expertise to respond / don’t know how to respond

AF23_3❑ 3 Health authorities do not want to respond

AF23_4❑ 4 Health authorities are too busy to respond

AF23_5❑ 5 It is not their responsibility to respond

AF23_6❑ 6 Other reason

**AF23sp1** Specify _______________________________________________________

**AF23sp2** Specify _______________________________________________________

**AF23sp3.** Specify _______________________________________________________

❑ 7 REFUSED

❑ 8 DON’T KNOW

**Avian Influenza Survey 12**

**AF24.** In a hospital, who should be responsible for reporting **epidemic infectious diseases** (for example, cholera) to government health authorities? **[DO NOT READ OUT RESPONSE OPTIONS]**

**[PROBE WITH EACH RESPONSE: “Anyone else?”]**

AF24_1❑ 1 Hospital authorities [Head of Department (HOD), Chief Medical Director (CMD), or hospital administrative leaders]

AF24_2❑ 2 Immediate supervisor/consultant

AF24_3❑ 3 Infectious disease consultant

AF24_4❑ 4 Hospital’s public health/community health department

AF24_5❑ 5 Infection control committee

❑ 6 Medical record officer (MRO)

❑ 7 A designated officer

**AF24officer**. Specify________________________________________________________

❑ 8 Physicians

❑ 9 Nurses

❑ 10 Laboratory technicians

❑ 11 NO one, since infectious diseases should not be reported to government authorities

❑ 12 Other

**AF24sp1** Specify ________________________________________________________

**AF24sp2** Specify ________________________________________________________

**AF24sp3.** Specify ________________________________________________________

❑ 97. REFUSED

❑ 98. DON’T KNOW

**AF25.** Should infectious diseases capable of causing an epidemic be reported to government health authorities?

(AF25) ❑ 1 Yes

❑ 2 NO

❑ 7 REFUSED

❑ 8 DON’T KNOW

**13 Avian Influenza Survey**

**AF26**. The previous questions referred to reporting epidemic infectious diseases. This question refers to **routine** **reporting** of notifiable diseases. In a hospital, who should be responsible for **routine** **reporting** of notifiable diseases to government health authorities)? **[DO NOT READ OUT RESPONSE OPTIONS]** **[PROBE WITH EACH RESPONSE: “Anyone else?”]**

AF26_1❑ 1 Hospital authorities [Head of Department (HOD), Chief Medical Director (CMD), or hospital administrative leaders]

AF26_2❑ 2 Immediate supervisor/consultant

AF26_3❑ 3 Infectious disease consultant

AF26_4❑ 4 Hospital’s public health/community health department

AF26_5❑ 5 Infection control committee

❑ 6 Medical record officer (MRO)

❑ 7 A designated officer

**AF26officer.** Specify:_____________________________________________________

❑ 8 Physicians

❑ 9 Nurses

❑ 10 Laboratory technicians

❑ 11 No one, since infectious diseases should not be reported to government authorities

❑ 12 I am not sure what the difference is between reporting epidemic infectious diseases and routine reporting of notifiable diseases.

❑ 13 Other

**AF26sp1** Specify _____________________________________________________

**AF26sp2** Specify _____________________________________________________

**AF26sp3.** Specify _____________________________________________________

❑ 97 REFUSED

❑ 98 DON’T KNOW

**Avian Influenza Survey 14**

**AF27.** Sometimes doctors may not report infectious disease to hospital authorities or government health authorities. What obstacles exist in this facility which may prevent a doctor from reporting infectious diseases to hospital authorities or to government health authorities? **[DO NOT READ OUT RESPONSE OPTIONS]** **[PROBE WITH EACH RESPONSE: “Any other reasons?”]**

AF27_1❑ 1 Doctors are too busy to report

AF27_2❑ 2 Doctors may not know that they should report / do not know the importance of reporting

AF27_3❑ 3 Doctors may not feel it is important to report or they may not care to report

AF27_4❑ 4 Doctors may want to protect the confidentiality of the patient with the disease

AF27_5❑ 5 Doctors don’t know which diseases to report

❑ 6 Doctors may feel the reporting process is too complicated or cumbersome

❑ 7 Doctors do not believe that reporting will lead to any response by the government authorities / nothing will be done / not taken seriously

❑ 8 Limited diagnostic or laboratory capacity

❑ 9 Doctor may believe that hospital management does not care or will take no action

❑ 10 Doctors do not believe it is their job to report / think that someone else is responsible for reporting

❑ 11 Doctors do not know how to report or to whom to report this information

❑ 12 Doctors may not have the appropriate materials for reporting (forms, telephone, etc)

❑ 13 Lack of infrastructure/logistics or reporting system

❑ 14 A doctor may not have ever seen an infectious disease that he/she was required to report

❑ 15 Other reason

**AF27sp.** Specify__________________________________________________________

❑ 97 REFUSED

15 = Other (specified in AF27sp1)

15 = No obstacles to report (specified in AF27sp1)

❑ 98 DON’T KNOW

**15 Avian Influenza Survey**

**AF28.** Now I am going to ask you about diseases that may or may not require reporting to government health authorities. For each disease, please tell me whether the disease requires **immediate reporting (within ½ day), routine reporting** or **does not require any reporting.**

|  | **Immediate reporting (within ½ day)** | **Routine** | **Does not require reporting** | **REFUSED** | **DON’T KNOW** |
| --- | --- | --- | --- | --- | --- |
| **AF28a.** AIDS | ❑ 1 | ❑ 2 | ❑ 3 | ❑ 7 | ❑ 8 |
| **AF28b.** Diarrhea | ❑ 1 | ❑ 2 | ❑ 3 | ❑ 7 | ❑ 8 |
| **AF28c.** Malaria | ❑ 1 | ❑ 2 | ❑ 3 | ❑ 7 | ❑ 8 |
| **AF28d.** Pneumonia | ❑ 1 | ❑ 2 | ❑ 3 | ❑ 7 | ❑ 8 |
| **AF28e.** Sexually Transmitted Disease | ❑ 1 | ❑ 2 | ❑ 3 | ❑ 7 | ❑ 8 |
| **AF28f.** Tuberculosis | ❑ 1 | ❑ 2 | ❑ 3 | ❑ 7 | ❑ 8 |
| **AF28g.** Polio/ Acute Flaccid Paralysis | ❑ 1 | ❑ 2 | ❑ 3 | ❑ 7 | ❑ 8 |
| **AF28h.** Dracunculiasis or Guinea Worm | ❑ 1 | ❑ 2 | ❑ 3 | ❑ 7 | ❑ 8 |
| **AF28i.** Leprosy | ❑ 1 | ❑ 2 | ❑ 3 | ❑ 7 | ❑ 8 |
| **AF28j.** Neonatal Tetanus | ❑ 1 | ❑ 2 | ❑ 3 | ❑ 7 | ❑ 8 |
| **AF28k.** Cholera | ❑ 1 | ❑ 2 | ❑ 3 | ❑ 7 | ❑ 8 |
| **AF28l.** Diarrhea with blood | ❑ 1 | ❑ 2 | ❑ 3 | ❑ 7 | ❑ 8 |
| **AF28m.** Measles | ❑ 1 | ❑ 2 | ❑ 3 | ❑ 7 | ❑ 8 |
| **AF28n.** Meningitis | ❑ 1 | ❑ 2 | ❑ 3 | ❑ 7 | ❑ 8 |
| **AF28o.** Plague | ❑ 1 | ❑ 2 | ❑ 3 | ❑ 7 | ❑ 8 |
| **AF28p.** Viral Hemorrhagic Fever (example: Lassa Fever) | ❑ 1 | ❑ 2 | ❑ 3 | ❑ 7 | ❑ 8 |
| **AF28q.** Avian Influenza or bird flu | ❑ 1 | ❑ 2 | ❑ 3 | ❑ 7 | ❑ 8 |
| **AF28r.** Yellow Fever | ❑ 1 | ❑ 2 | ❑ 3 | ❑ 7 | ❑ 8 |

**AF29.** During which stage of your medical training did you **first** learn about disease surveillance or

(AF29) disease notification to public health authorities? **[READ OUT RESPONSE OPTIONS]**

❑ 1 Medical school

❑ 2 Internship or residency

❑ 3 Subspecialty training

❑ 4 Master’s training

❑ 5 Other

**AF29sp**. Specify:_________________________________________________________

❑ 6 Never learned about notifiable infectious diseases during my medical training

❑ 7 REFUSED

❑ 8 Don’t know

**Avian Influenza Survey 16**

**SECTION D: Sources of Information and Communication**

Now I am going to ask you about your sources of health information and ways you communicate.

1 = box marked

2 = no mark in box

7 = “97” marked (Refused)

8 = “98” marked (Don’t Know)

**AF30.** How do you **usually** learn about health information?

**[DO NOT READ OUT RESPONSE OPTIONS]**

**[PROBE WITH EACH RESPONSE: “Any other ways?”]**

AF30_1❑ 1 Educational lecture or course

AF30_2❑ 2 Training sessions/seminars/workshops

AF30_3❑ 3 State or federal ministry of health communication (for example: letter, bulletin, or newsletter)

AF30_4❑ 4 Medical book

AF30_5❑ 5 Medical journal

❑ 6 Medical school/or postgraduate medical training (for example: internship, residency, or fellowship)

❑ 7 Professional colleagues

❑ 8 Pamphlets

❑ 9 Posters

❑ 10 Billboards

❑ 11 Internet website

**AF30web.** Specify website _________________________________________________

❑ 12 Email

❑ 13 Newspaper/magazines

❑ 14 Radio

❑ 15 Television

❑ 16 Cell phone text message or short message service (SMS)

❑ 17 Public lecture or through community enlightenment

❑ 18 Neighbors/friends

❑ 19 Religious or traditional leader (tribal, village, or community leader)

❑ 20 Other Source

**AF30sp1** Specify ______________________________________________________

**AF30sp2** Specify ______________________________________________________

**AF30sp3.** Specify ______________________________________________________

❑ 97 Refused

❑ 98 Don’t Know

**AF31.** What are the best ways to get health information to you?

**[DO NOT READ OUT RESPONSE OPTIONS]**

**[PROBE ONLY ONCE AFTER FIRST RESPONSE: “Any other ways?”]**

AF31_1❑ 1 Educational lecture or course

AF31_2❑ 2 Training sessions/seminars/workshops

AF31_3❑ 3 State or federal ministry of health communication (for example: letter, bulletin, or newsletter)

AF31_4❑ 4 Medical book

AF31_5❑ 5 Medical journal

❑ 6 Medical school/or postgraduate medical training (for example: internship, residency, or fellowship)

❑ 7 Professional colleagues

❑ 8 Pamphlets

❑ 9 Posters

❑ 10 Billboards

❑ 11 Internet website

**AF31web**. Specify website ________________________________________________

❑ 12 Email

❑ 13 Newspaper/magazines

❑ 14 Radio

❑ 15 Television

❑ 16 Cell phone text message or short message service (SMS)

❑ 17 Public lecture or through community enlightenment

❑ 18 Neighbors/friends

❑ 19 Religious or traditional leader (tribal, village, or community leader)

❑ 20 Other source

**AF31sp1.** Specify ______________________________________________________

**AF31sp2.** Specify ______________________________________________________

**AF31sp3**. Specify ______________________________________________________

❑ 97 Refused

❑ 98 Don’t Know

**17 Avian Influenza Survey**

**Avian Influenza Survey 18**

On a scale of 1 to 5, with 1 meaning “absolutely **do not** believe” and 5 meaning “**absolutely believe**,” how much would you believe health information from the following sources?

|  | **1**  **Absolutely**  **do not believe** | **2**  **Somewhat**  **do not believe** | **3**  **Neutral** | **4**  **Somewhat believe** | **5**  **Absolutely believe** | **REFUSED** | **DON’T KNOW** |
| --- | --- | --- | --- | --- | --- | --- | --- |
| **AF32.** TV | ❑ 1 | ❑ 2 | ❑ 3 | ❑ 4 | ❑ 5 | ❑ 7 | ❑ 8 |
| **AF33.** Radio | ❑ 1 | ❑ 2 | ❑ 3 | ❑ 4 | ❑ 5 | ❑ 7 | ❑ 8 |
| **AF34.** Newspaper | ❑ 1 | ❑ 2 | ❑ 3 | ❑ 4 | ❑ 5 | ❑ 7 | ❑ 8 |
| **AF35.** İnternet websites besides those of the government or WHO | ❑ 1 | ❑ 2 | ❑ 3 | ❑ 4 | ❑ 5 | ❑ 7 | ❑ 8 |
| **AF36.** Professional colleagues | ❑ 1 | ❑ 2 | ❑ 3 | ❑ 4 | ❑ 5 | ❑ 7 | ❑ 8 |
| **AF37.** Hospital authorities | ❑ 1 | ❑ 2 | ❑ 3 | ❑ 4 | ❑ 5 | ❑ 7 | ❑ 8 |
| **AF38.** WHO or UNICEF | ❑ 1 | ❑ 2 | ❑ 3 | ❑ 4 | ❑ 5 | ❑ 7 | ❑ 8 |
| **AF39.** CDC | ❑ 1 | ❑ 2 | ❑ 3 | ❑ 4 | ❑ 5 | ❑ 7 | ❑ 8 |
| **AF40.** Government health authorities | ❑ 1 | ❑ 2 | ❑ 3 | ❑ 4 | ❑ 5 | ❑ 7 | ❑ 8 |
| **AF41.** Religious or traditional leaders | ❑ 1 | ❑ 2 | ❑ 3 | ❑ 4 | ❑ 5 | ❑ 7 | ❑ 8 |
| **AF42.** NGOs | ❑ 1 | ❑ 2 | ❑ 3 | ❑ 4 | ❑ 5 | ❑ 7 | ❑ 8 |

**AF43.** How often do you watch TV?

**[READ OUT RESPONSE OPTIONS][ONLY ONE ANSWER IS POSSIBLE]**

❑ 1 At least once per day

❑ 2 At least once per week

❑ 3 At least once per month

❑ 4 Less than once per month

❑ 5 Never

❑ 7 Refused

❑ 8 Don’t Know

**19 Avian Influenza Survey**

**AF44.** How often do you listen to the radio?

**[READ OUT RESPONSE OPTIONS][ONLY ONE ANSWER IS POSSIBLE]**

❑ 1 At least once per day

❑ 2 At least once per week

❑ 3 At least once per month

❑ 4 Less than once per month

❑ 5 Never

❑ 7 REFUSED

❑ 8 Don’t Know

**AF45.** Do you have a cell phone that you currently use to make and receive calls?

❑ 1 Yes

❑ 2 NO

**IF “NO”, “REFUSED”, or “DON’T KNOW”**

**GO TO QUESTION AF47.**

❑ 7 REFUSED

❑ 8 DON’T KNOW

**AF46.** How often do you send or receive text messages on your cell phone?

**[READ OUT RESPONSE OPTIONS][ONLY ONE ANSWER IS POSSIBLE]**

❑ 1 At least once per day

❑ 2 At least once per week

❑ 3 At least once per month

❑ 4 Less than once per month

❑ 5 Never

❑ 7 Refused

❑ 8 Don’t Know

**AF47.** How often do you use the internet?

**[READ OUT RESPONSE OPTIONS] [ONLY ONE ANSWER IS POSSIBLE]**

❑ 1 At least once per day

❑ 2 At least once per week

❑ 3 At least once per month

❑ 4 Less than once per month

❑ 5 Never

**IF “Never”, “REFUSED”, or “DON’T KNOW”**

**GO TO QUESTION AF50.**

❑ 7 Refused

❑ 8 Don’t Know

**IF AF47 = “1”, “2”, “3” or “4” THEN ASK AF48 AND AF49**

**AF48.** When you use the internet, where do you usually access the internet?

**[DO NOT READ OUT RESPONSE OPTIONS] [ONLY ONE ANSWER IS POSSIBLE]**

❑ 1 At home

❑ 2 At the hospital

❑ 3 At a public internet café

5 = Other (specified in AF48sp)

5 = More than one answer marked

(numeric code of responses recorded in AF48sp)

❑ 4 At a friend’s house

❑ 5 Other place

**AF48sp.** Specify:_______________________________________________________

❑ 7 Refused

❑ 8 Don’t Know

**AF49.** How often do you look up medical information on the internet?

**Avian Influenza Survey 20**

**[READ OUT RESPONSE OPTIONS][ONLY ONE ANSWER IS POSSIBLE]**

❑ 1 At least once per day

❑ 2 At least once per week

❑ 3 At least once per month

❑ 4 Less than once per month

❑ 5 Never

❑ 7 Refused

❑ 8 Don’t Know

**AF50.** How often do you read medical journals? This can be either hard copies or online versions.

**[READ OUT RESPONSE OPTIONS][ONLY ONE ANSWER IS POSSIBLE]**

❑ 1 At least once per day

❑ 2 At least once per week

❑ 3 At least once per month

❑ 4 Less than once per month

❑ 5 Never

❑ 7 REFUSED

❑ 8 Don’t Know

**21 Avian Influenza Survey**

**AF51.** Have you read any medical journals published in the past three months? This can be either hard copies or (AF51) online versions. **[DO NOT READ OUT RESPONSE OPTIONS]**

❑ 1 YES

Please specify which three you read most often:

**AF51sp1** 1) ___________________________________________________________

**AF51sp2** 2) ___________________________________________________________

**AF51sp3.** 3) ___________________________________________________________

❑ 2 NO

❑ 7 Refused

❑ 8 Don’t Know

**AF52.** How often do you attend medical lectures at your hospital?

**[READ OUT RESPONSE OPTIONS][ONLY ONE ANSWER IS POSSIBLE]**

❑ 1 At least once per day

❑ 2 At least once per week

❑ 3 At least once per month

❑ 4 Less than once per month

❑ 5 Never

❑ 6 My hospital does not give medical lectures

❑ 7 Refused

❑ 8 Don’t Know

**Avian Influenza Survey 22**

**AF53.** How often do you attend medical conferences or medical courses?

**[READ OUT RESPONSE OPTIONS][ONLY ONE ANSWER IS POSSIBLE]**

❑ 1 At least once per day

❑ 2 At least once per week

❑ 3 At least once per month

❑ 4 At least once every six months

❑ 5 At least once every year

❑ 6 At least once every two years

❑ 7 Less than every two years

❑ 8 Never

❑ 97 Refused

❑ 98 Don’t Know

**Avian Influenza Survey 22**

**AF54.** Are you required to complete continuing medical education (CME) to maintain your medical license?

❑ 1 Yes

❑ 2 NO

❑ 7 Refused

❑ 8 Don’t Know

**AF55.** In what types of continuing medical education (CME) do you usually participate?

**[DO NOT READ OUT RESPONSE OPTIONS]**

**[PROBE ONLY ONCE AFTER FIRST RESPONSE: “Any other ways?”]**

AF55_1❑ 1 Educational lecture or course

1 = box marked

2 = no mark in box

7 = “97” marked (Refused)

8 = “98” marked (Don’t Know)

AF55_2❑ 2 Training sessions/seminars/workshops/conferences

AF55_3❑ 3 State or federal ministry of health communication (for example: letter, bulletin, or newsletter)

AF55_4❑ 4 Medical book

AF55_5❑ 5 Medical journal

AF55_6❑ 6 Medical internet website

**AF55web.** Specify website ________________________________________________

AF55_7❑ 7 Other source

**AF55sp1** Specify ______________________________________________________

**AF55sp2** Specify ______________________________________________________

**AF55sp3.** Specify ______________________________________________________

AF55_8❑ 8 I do not participate in continuing medical education

❑ 97 Refused

❑ 98 Don’t Know

**AF56.** Do you have an email address?

❑ 1 YES

❑ 2 NO

**IF “NO”, “REFUSED”, or “DON’T KNOW”**

**GO TO SECTION E, QUESTION AF58 ON PAGE 23.**

❑ 7 REFUSED

❑ 8 DON’T KNOW

**AF57.** How often do you check your email?

**[READ OUT RESPONSE OPTIONS][ONLY ONE ANSWER IS POSSIBLE]**

❑ 1 At least once per day

❑ 2 At least once per week

❑ 3 At least once per month

❑ 4 Less than once per month

❑ 5 Never

❑ 7 Refused

❑ 8 Don’t Know

**23 Avian Influenza Survey**

**SECTION E: Demographics**

Finally, I'd like to ask some questions about you and your background.

**AF58.** How old are you?

Years

❑ 97 Refused

❑ 98 Don’t Know

**AF59.** What is your first native language? **[ONLY ONE ANSWER IS POSSIBLE]**

❑ 1 English

❑ 2 Hausa

❑ 3 Igbo

❑ 4 Yoruba

❑ 5 Other

**AF59sp.** Specify________________________________________________________

❑ 7 Refused

❑ 8. Don’t Know

**AF60.** What is the highest level of medical education you have completed?

**[READ RESPONSE OPTIONS] [ONLY ONE ANSWER IS POSSIBLE]**

❑ 1 Medical school only

❑ 2 Medical Housemanship or Internship

❑ 3 Medical residency, fellowship, or subspecialty training

❑ 4 Other

**AF60sp.** Specify ________________________________________________________

❑ 7 Refused

❑ 8 Don’t Know

**AF61.** For your medical training, did you study outside or in Nigeria?

**[READ RESPONSE OPTIONS] [ONLY ONE ANSWER IS POSSIBLE]**

❑ 1 Outside only

❑ 2 In Nigeria only

❑ 3 Both outside and in Nigeria

❑ 7 Refused

❑ 8 Don’t Know

**Avian Influenza Survey 24**

**AF62.** In what clinical capacity do you work in this facility? **[READ RESPONSE OPTIONS]**

❑ 1 General practice

❑ 2 Family practice

❑ 3 Pediatrics

❑ 4 Internal medicine

❑ 5 Public health/community medicine

7 = Other (specified in AF62sp)

7 = More than one answer marked

(numeric code of responses recorded in AF62sp)

❑ 6 Emergency medicine

❑ 7 Other

**AF62sp.** Specify: ________________________________________________________

❑ 97 Refused

❑ 98 Don’t Know

**AF63.** Including internship and residency, how many years have you been practicing clinical medicine?

Years

❑ 97 Refused

❑ 98 Don’t Know

**AF64.** In the past three months have you received clinical supervision from your direct supervisor as part of your clinical work?

❑ 1 Yes

❑ 2 NO

**IF “NO”, “REFUSED”, or “DON’T KNOW” GO TO END.**

❑ 7 REFUSED

❑ 8 DON’T KNOW

**AF65.** In the past three months how often did you receive clinical supervision from you direct supervisor?

**[READ RESPONSE OPTIONS]** **[ONLY ONE ANSWER IS POSSIBLE]**

❑ 1 Daily

❑ 2 At least once weekly

❑ 3 At least once monthly

❑ 4 Rarely or never

❑ 7 Refused

❑ 8 Don’t Know

**END**. That’s the end of the interview. Thank you for contributing to this important study.

**25 Avian Influenza Survey**

Entered in separate (Excel) file

**AFremark**. Please write any remarks and clarifications here.

section not entered

DAY MONTH YEAR

**/**

**/**

Reviewed by: ❑ Field Supervisor Date:

DAY MONTH YEAR

**/**

**/**

❑ Field Research Manger Date:

DAY MONTH YEAR

**/**

**/**

❑ Field Monitor Date
